# Sleep Spindle Abnormalities in Preschool Children With Autism Spectrum Disability: Insights From Nap Polysomnography

**DOI:** 10.1002/aur.70087

**Published:** 2025-07-23

**Authors:** Sasha D’Ambrosio, Daniele Gualandris, Davide Caputo, Francesco Donati, Ahmad Mayeli, Renata del Giudice, Fabio Ferrarelli, Alessia Mingarelli, Federico Raviglione, Maria Paola Canevini, Armando D’Agostino

**Affiliations:** ^1^ Department of Health Sciences Università degli Studi di Milano Milan Italy; ^2^ Department of Clinical and Experimental Epilepsy University College London London UK; ^3^ IRCCS Fondazione Don Carlo Gnocchi, ONLUS Milan Italy; ^4^ Department of Mental Health and Addiction ASST Brianza Vimercate Italy; ^5^ Department of Pediatric Neuroscience Fondazione IRCCS Istituto Neurologico Carlo Besta Milan Italy; ^6^ Department of Psychiatry University of Pittsburgh Pittsburgh USA; ^7^ Department of Mental Health and Addiction ASST Santi Paolo e Carlo Milan Italy; ^8^ Childhood and Adolescence Neuropsychiatry Unit Ospedale “G. Salvini” Rho Italy

## Abstract

Sigma power and sleep spindles are key elements of Non‐Rapid Eye Movement (NREM) sleep. They reflect anatomical and physiological properties of brain circuits, are linked with various behavioral outcomes in typically development (TD) children, and undergo significant modifications during development. Furthermore, recent studies have highlighted the potential of NREM sigma power and sleep spindles as early neurophysiological markers for autism spectrum disability (ASD). Here, we conducted polysomnography (PSG)/EEG recordings during afternoon naps on 50 children aged between 2 and 6 years, diagnosed with ASD or TD. EEG recordings from 19 scalp leads were analyzed, focusing on sigma power and sleep spindle parameters. EEG analyses revealed significant differences in power spectral density between ASD and TD children, particularly in the sigma band and adjacent alpha and beta bands, with increased power localized to anterior EEG leads in ASD children. Higher spindle amplitude and integrated spindle activity (ISA) were found in the ASD group, especially in frontal regions. Additional frequency‐specific analyses (10–12 Hz, 12–14 Hz, 14–16 Hz) confirmed significant differences in spindle amplitude and distribution patterns, emphasizing the role of brain regions that are detectable from anterior EEG leads in ASD‐related sleep abnormalities. No significant differences were found in spindle density, duration, or frequency outside specific clusters. These findings indicate that some sleep spindle parameters, particularly in frontal areas, are altered in ASD. The study highlights the feasibility of using afternoon nap PSG as a practical and effective method to detect these abnormalities in clinical settings. Future research should investigate the developmental trajectory of spindles in ASD and their potential role as neurophysiological biomarkers, offering valuable insights for diagnosis and prognosis.

**Summary**

- This study examines differences in specific sleep EEG patterns between children on the autism spectrum and typically developing children.
- We found significant variations in the frontal brain regions of children on the autism spectrum that may be relevant for cognitive and socio‐communicative development.
- These findings broaden our understanding of autism and could enhance diagnostic and prognostic methods that may be crucial for both scientific and medical communities.

## 1 Introduction

Brain oscillatory activity during NREM sleep, encompassing phenomena such as sleep spindles and slow waves, reflects anatomical and physiological properties of brain circuits.

Sleep spindles, which characterize NREM sleep stages 2 (N2), undergo substantial modifications during cerebral maturation, reflecting a critical role for spindle oscillations in brain development (Gorgoni et al. 2020). In typical development (TD), these NREM features have been associated not only with anatomical substrates, but also with behavioral outcomes such as intelligence (Geiger et al. 2011), visual perception (Bang et al. 2014), memory (Chatburn et al. 2013; Fogel et al. 2007), motor skills (Kurth et al. 2012; Lustenberger et al. 2017), language, social, and cognitive functioning (Page et al. 2018). Furthermore, the maturation of the thalamocortical network in TD is associated with the evolution of sleep spindle patterns across various brain regions. For example, the premature occurrence of slow frontal spindles has been demonstrated to precede the onset of rapid centro‐parietal spindles (see Clawson et al. 2016 for a review on this topic). A machine learning study of polysomnographic (PSG) data recently showed that sleep spindle density, amplitude, and spindle‐slow oscillation (SSO) coupling emerged as key discriminative features between typical development and autism spectrum development, along with aperiodic signal characteristics and the percentage of REM sleep (Martinez and Chen 2023). While spindles have been associated with learning in TD (Gorgoni et al. 2020), their relationship with intellectual abilities in children and adolescents remains unclear (Gorgoni et al. 2020; Gruber et al. 2016).

A recent study of daytime naps in 13‐ to 30‐month‐old children on the autism spectrum revealed topographically distinct decreases in fast theta (5–7.25 Hz) and fast sigma (15–16 Hz) oscillations, and increased beta (20–25 Hz) oscillations compared to age‐matched TD children (Page et al. 2020). Furthermore, a large cohort study found reduced spindle density in young children with autism spectrum disability (ASD) relative to age‐matched children with developmental delay (DD) or TD (Farmer et al. 2018). In this work, no significant differences were found between TD and DD groups for any spindle parameters. Mylonas et al. (2022) also reported a spindle density deficit during N3 sleep in 19 children with ASD. However, one sample of 14 children displayed a broader distribution of spindle density compared to the TD sample (Kawahara et al. 2022), with five children on the autism spectrum showing higher spindle density. Furthermore, sleep architecture, spindles, and power in sleep‐stage‐specific EEG frequency bands were found to be remarkably comparable between 22 children with “high‐functioning” ASD and a TD sample (Kurz et al. 2019), suggesting impaired spindle activity may be more prominent in ASD children with the co‐occurrence of DD.

Overall, the relationship between impaired spindle activity and the DD in ASD is still poorly understood. Furthermore, there is a shortage of sleep studies that have examined sigma power and sleep spindle parameters, including their topographic characteristics, in ASD children with and without DD. Therefore, in the current study, we aimed to expand on these findings by investigating the parameters and topography of sleep spindles in children on the autism spectrum with the different developmental features that can be observed in naturalistic clinical contexts, including DD and/or epileptiform abnormalities. Given the challenges of conducting comprehensive overnight PSG in various clinical environments, we aimed to validate the microstructural characterization of spindle oscillations during afternoon naps, which are more feasible in standard clinical practice. Hence, our work aims to elucidate the key role of sigma power sleep spindle parameters in ASD and underscore the potential of sleep spindle analysis as a diagnostic and prognostic tool in ASD, particularly in clinical settings where comprehensive overnight PSG is challenging.

## 2 Methods

### 2.1 Subjects

We recorded PSG with 19 scalp EEG leads during afternoon naps from 50 children between 2 and 6 years of age diagnosed with ASD according to DSM‐5 criteria (Diagnostic and statistical manual of mental disorders 2013) or with TD. Depending on age and language level, clinical diagnosis was integrated with ADOS test (Autism Diagnostic Observation Schedule; Toddler module or module 1 or module 2). Cognitive abilities were assessed with Griffiths or Wechsler Scales (Griffiths 1970; Wechsler 2012). In accordance with clinical assessment, we established three exploratory subgroups within the ASD cohort: children with autism spectrum diagnosis only (ASDo); with co‐occurring General Developmental Delay (ASD‐GDD); and with both GDD and EEG epileptic abnormalities (ASD‐GDD‐E, without clinical history of epilepsy and free from EEG seizures). Only participants who reached sleep stages N2 and N3 were included. We excluded children on the autism spectrum with a clinical history of febrile seizures, epilepsy, Central Nervous System (CNS) malformations, extreme chronotypes, and those who were taking medication. To ensure appropriate control comparisons, we matched TD controls to ASD children based on age and sex. In conducting this study, we adhered to the principles outlined in the Declaration of Helsinki regarding the ethical conduct of research involving human subjects. All procedures were performed in accordance with these principles to ensure the protection of participants' rights, confidentiality, and welfare.

### 2.2 EEG Recording and Visual Scoring

Nap EEGs were recorded using a standard 20‐electrodes EEG system (System Plus Mogliano Tv, Italy). EEG signal was referenced to the Fz electrode and recorded at a sampling rate of 256 Hz. Nineteen electrodes were positioned using the 10:20 international system. All electrodes' impedances were kept below 5 KΩ. Napping was spontaneous for all participants except one, for whom sleep was pharmacologically induced with promazine 1 mg/kg. Two co‐authors (D.G., D.C.) independently visually inspected the EEG activity to exclude participants with relevant artifacts. EEGs with isolated, sporadic (less than 25% of an epoch) epileptic abnormalities that did not interfere with the organization of sleep (i.e., isolated spikes) were included. Sleep data from 6 EEG electrodes (F3/FP10, F4/FP9, C3/FP10, C4/FP9, O1/FP10, O2/FP9) were segmented in 30 s epochs and visually scored by one experienced EEG specialist (D.C.) following the American Academy of Sleep Medicine (AASM) criteria (Silber et al. 2007).

### 2.3 EEG Signal Analysis

We employed Matlab (The MathWorks Inc., Natick, MA) to develop an original pipeline for the analysis of EEG recordings using functions from the Matlab‐based public license toolbox EEGLAB (Delorme and Makeig 2004). First, we applied a band‐pass filter (1.6–70 Hz) on the EEG signals. We then rejected electrodes and epochs with large artefactual activity using a previously reported semi‐automatic procedure based on thresholds of mean power for low (1–4 Hz) and high (20–30 Hz) frequency ranges for each electrode (following the same rational as in Huber et al. 2000). EEG signal was then re‐referenced to the average of the scalp voltage of all 19 electrodes and split into six‐second epochs. Spectral power in each frequency range across all six‐second NREM epochs was plotted and visually inspected for each electrode. Moreover, Welch's averaged modified periodogram with a Hamming window was employed to decompose the resulting EEG signal into the frequency domain. Finally, EEG power spectra were compared between ASD and TD groups in the following five frequency bands: delta (1–4.5 Hz); theta (4.5–8 Hz); alpha (8–11.5 Hz); sigma (11.5–15.5 Hz) high beta (15–25 Hz); and gamma (25–40 Hz).

### 2.4 Spindle Detection

We adopted automated procedures similar to those reported in previous studies for the detection of sleep spindles (D’Agostino et al. 2018; Ferrarelli et al. 2012, Ferrarelli et al. 2010; Kaskie et al. 2019). NREM epochs were filtered between 10 and 16 Hz, and the rectified filtered traces were used as time series for each electrode. Thresholds for spindle detection were set between two and six times the mean amplitude of each electrode. An amplitude fluctuation exceeding the upper threshold determined the detection of a spindle event. Time points around this peak where signal amplitude dropped below the lower threshold marked the beginning and end of a spindle. We calculated the following sleep spindle parameters: amplitude, duration, density (i.e., number of spindles per minute), mean frequency, and integrated spindle activity (ISA). Importantly, ISA was calculated by integrating the absolute amplitude values of each detected spindle at every electrode, then dividing this sum by the total nap duration. This metric provides an overall measure of spindle activity intensity relative to the length of the sleep period, reflecting the density and strength of spindle occurrences during the recording. Furthermore, similarly to previous literature in children (Chatburn et al. 2013; Lustenberger et al. 2015), we characterized spindle activity in different sub‐ranges based on their frequency (similarly to Gonzalez et al. 2022, Gonzalez et al. 2018; Timofeev and Chauvette 2013), specifically, 10–12 Hz (very slow), 12–14 Hz (slow), 14–16 Hz (fast). Dividing spindle activity into these sub‐frequency bands is essential for capturing distinct functional roles that different spindle frequencies may play in neurocognitive and sensorimotor processes. Each frequency range could reflect unique neural mechanisms or developmental differences in spindle generation, which are particularly relevant when studying children. Spindle parameters were computed for each of these frequency ranges and compared between groups.

### 2.5 Statistical Analysis

In this study, we compared EEG power spectra and spindles parameters between the two groups of interest (overall ASD vs. TD) using the cluster‐based permutation test provided by the FieldTrip toolbox (Oostenveld et al. 2011). After preprocessing, we spatially prepared data using FieldTrip functions *ft_prepare_neighbours* for neighbor configuration. We used FieldTrip's non‐parametric cluster‐based permutation test for comparisons between groups for every parameter of interest using the following configuration: cfg.method was set to “montecarlo,” implementing a Monte Carlo permutation approach; cfg.statistic was chosen as “indepsamplesT” for independent samples *T*‐test; cluster correction was applied (cfg.correctm = “cluster”), with a cluster‐forming threshold (cfg.clusteralpha = 0.05) and a cluster significance threshold (cfg.clusterstatistic = “maxsum”). The minimum number of neighboring channels required to form a cluster was set to two (cfg.minnbchan = 2), with a significance level (cfg.alpha = 0.05) and the number of random permutations (cfg.numrandomization = 1000) was selected to balance type I and II error rates. Moreover, we further compared the statistically significant clusters between the two groups and presented the distribution of the mean values from these clusters by means of a non‐parametric Mann–Whitney *U* test. We computed effect sizes using Cohen's *d*, defined as the difference between the two group means divided by their pooled standard deviation. Power was estimated through MATLAB's sampsizepwr function based on the effect size, sample sizes, and an alpha level of 0.05 (detailed results are listed in the Tables S2–S4). Of note, throughout the entire manuscript *p* values have two decimal places, values under 0.01 are represented as *p* < 0.01 and considered statistically significant if lower or equals to 0.05. Analyses involving the ASD subgroups (ASDo, ASD‐GDD, ASD‐GDD‐E) were exploratory and descriptive. These subgroup comparisons were conducted to visualize potential trends and generate hypotheses for future studies, but were not subjected to formal inferential testing.

### 2.6 Computational Analysis Functions

For preprocessing analysis, quantifications, comparisons and visualization, we used original Matlab scripts (The MathWorks Inc., version R2018b), incorporating EEGLAB and FieldTrip toolboxes. Specifically, for preprocessing: *pop_select* (E), *filtfilt* (E), *pop_reref* (E), *pwelch* (M), *ft_prepare_neighbours* (F), *ft_channelrepair with “spline method”* (F); For quantification and statistics: *ranksum* (M), sampsizepwr, *ft_timelockstatistics with cfg.method = “montecarlo*” (F), *cfg.statistic = “indepsamplesT*” (F), *cfg.correctm =* “*cluster*” (F), *cfg.clusteralpha = 0.05, cfg.clusterstatistic =* “*maxsum*” (F), *cfg.minnbchan = 2* (F); *cfg.alpha = 0.05, cfg.numrandomization = 1000* (F); For representation: *boxplot* (M), *topoplot* (E), *ft_topoplotER* (F).

## 3 Results

### 3.1 Subgroup Demographics

A total of 32 children with ASD (3 females; mean age: 3.77 ± 0.97 years) and 18 children with TD (7 females; mean age: 3.37 ± 1.23 years) were recruited (Table 1, single subject information and clinical data can be found in Table S1). These two groups did not differ in terms of age (*p* = 0.53), whereas males were found to be significantly more frequent in children on the autism spectrum (*p* = 0.024). Furthermore, the number of children gender ratio, and age are reported below for the ASD subgroups used for descriptive purposes.

**TABLE 1:** (1) ASDo, 6 children (no females, mean age: 4.06 ± 1.43 years); (2) ASD‐GDD, 19 children (16 males and 3 females, mean age: 3.56 ± 0.81 years); (3) ASD‐GDD‐E, 7 male children (mean age: 4.09 ± 0.93 years).

	ASD	TD	ASDo	ASD‐GDD	ASD‐GDD‐E
Number	32	18	6	19	7
Gender M:F	3:29	7:11	6:0	16:3	7:0
Age	3.77	3.37	4.06	3.56	4.09
(s.d.)	(0.97)	(1.23)	(1.43)	(0.81)	(0.93)

*Note*: Demographic data with differences between groups. This table presents the number of subjects, gender distribution (M:F), mean age (with standard deviation, s.d.). The subgroups included: ASDo (children with ASD only), ASD‐GDD (children with ASD and General Developmental Delay), and ASD‐GDD‐E (children with ASD, General Developmental Delay, and EEG epileptic abnormalities).

### 3.2 Sleep Architecture

The TD group showed a significantly longer mean total sleep time (TST) when compared with the ASD group (44.60 ± 13.48 vs. 35.19 ± 11.66, *p* = 0.01), and the ASD‐GDD‐E group (44.60 ± 13.48 vs. 34.89 ± 12.55, *p* = 0.03). No difference was observed for mean sleep duration and percentage of N1, N2, and N3 phases during NREM sleep.

### 3.3 EEG Power Spectra Comparisons

**FIGURE 1:**
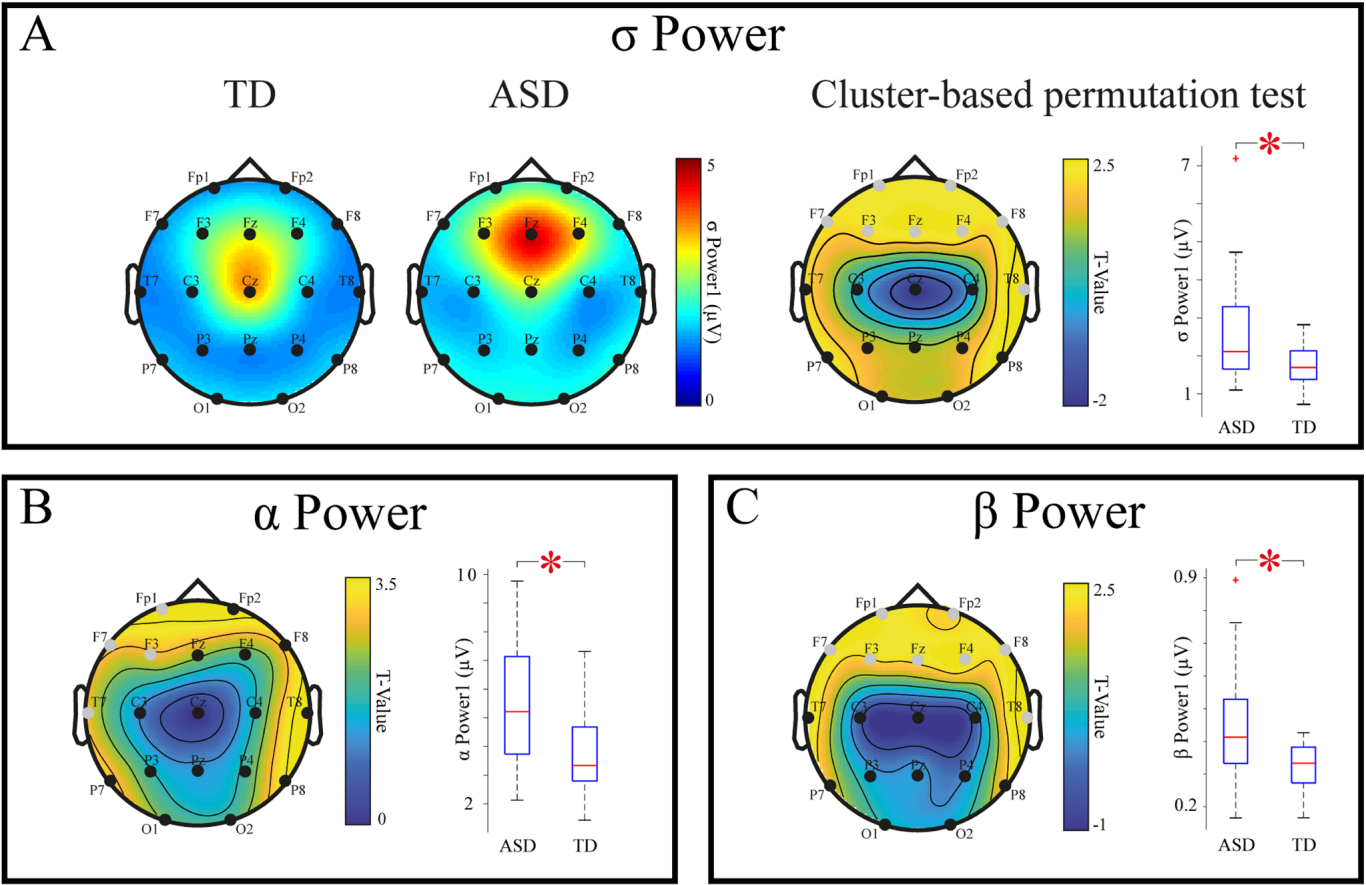
Power spectrum in children on the autism spectrum. Panel (A) Left, Topographic distribution of sigma (*σ*) power in TD and ASD children. Centre, topographic map of *T*‐values derived from *t*‐tests for independent samples within a cluster‐based permutation test, comparing ASD and TD groups. On the right, boxplots display the distribution of mean values averaged within the statistically significant clusters identified. Panel (B) Left, topographic maps of the cluster‐based permutation test *T*‐Values for comparing alpha (*α*) power between ASD and TD groups. Right, boxplot displays the distribution of mean values averaged within the statistically significant clusters identified. Panel (C) Similar to panel B for beta (*β*) power comparisons. In all panels, the Mann–Whitney *U* test is utilized for comparing distribution means (boxplot), with asterisks indicating statistical significance. Gray EEG leads on the topographic maps denote channels that are part of a statistically significant cluster.

Comparative analyses of power spectral density between ASD and TD children consistently revealed differences within the frequency range associated with sleep spindles, namely within the sigma band and the adjacent alpha and beta bands. Whereas no differences were found among comparisons of Delta, Theta, and Gamma bands. Statistically significant power increases were observed exclusively in anterior EEG leads. Specifically, statistical analysis revealed increased power in a cluster composed of bilateral frontal electrodes and the T8 electrode in the sigma band (Figure 1A, Mann–Whitney *U* (*U*) = 927, *p* = 0.02), in a left frontal cluster in the alpha band (Figure 1B, *U* = 967, *p* = 0.01), and in a cluster composed of bilateral frontal electrodes and the T8 electrode in the beta band (Figure 1C, *U* = 943, *p* = 0.01). Please refer to Figure S1 and Table S2 for detailed and broad band results.

### 3.4 Sleep Spindles

We found statistically significant differences in spindle parameters when comparing children on the autism spectrum and TD children. We calculated spindle parameters both in a broad frequency range between 10 and 16 Hz (for detailed results refer to Table S3), and in three frequency sub‐ranges. Namely, (a) 10–12 Hz, (b) 12–14 Hz, and (c) 14–16 Hz (detailed results in Table S4).

### 3.5 Comparisons of Broad Band Sleep Spindles

**FIGURE 2:**
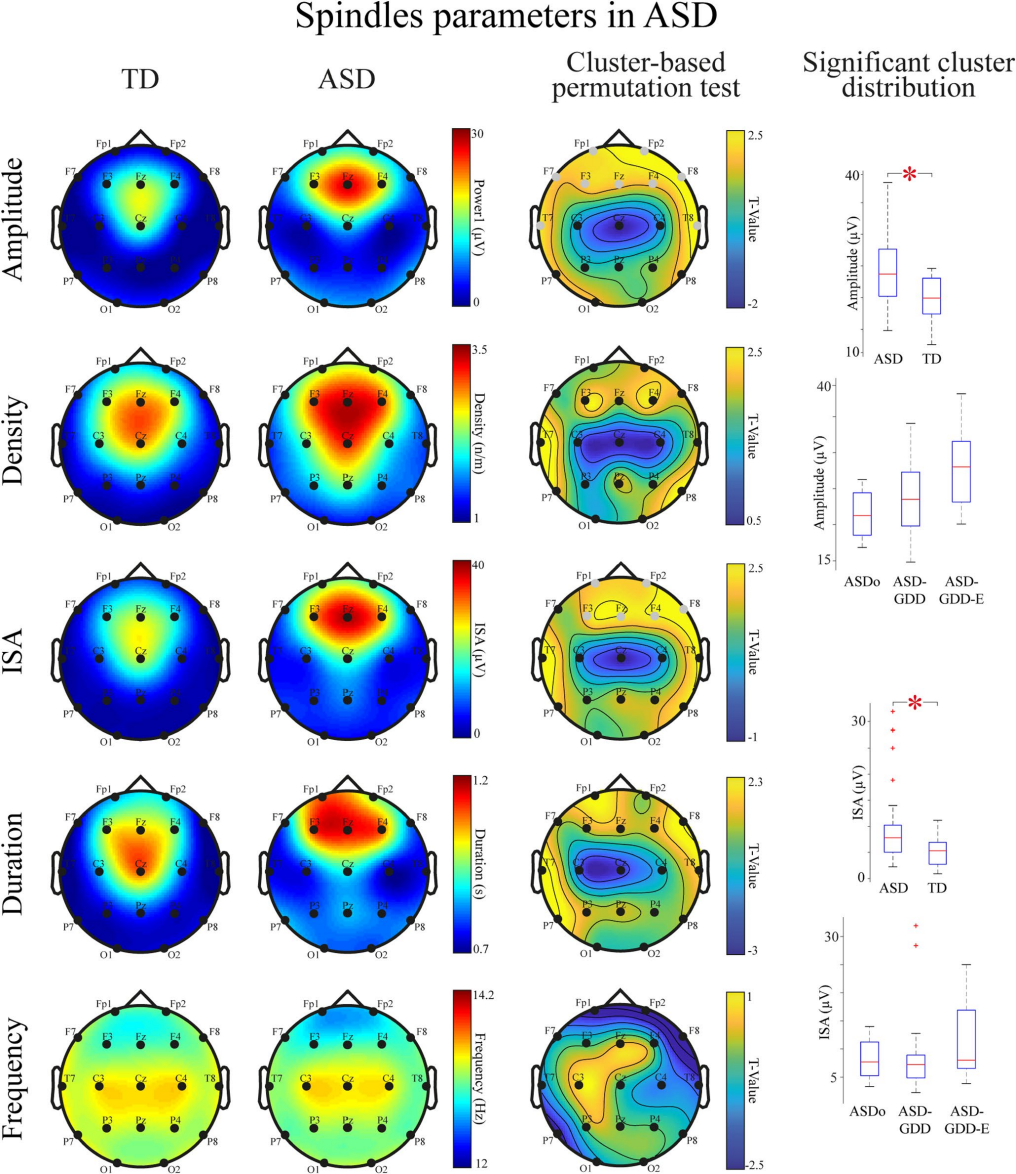
Spindle parameters in children on the autism spectrum. Each row displays spindle parameters for TD and ASD children, presented in the first and second columns, respectively. The third column in each row features topographic maps showing *T*‐values derived from *t*‐tests for independent samples, applied within a cluster‐based permutation test to compare the two groups. The parameters analyzed from top to bottom are Amplitude, Density, ISA, Duration, and Frequency. On the right, the final column highlights the two parameters that showed significant differences in the cluster‐based permutation test: Amplitude and ISA. For these parameters, two boxplots illustrate the distribution of mean values averaged within the statistically significant clusters. Additionally, under each of these distributions three descriptive boxplots display the distribution of mean values within significant clusters identified among ASD subgroups, specifically ASDo, ASD‐GDD, and ASD‐GDD‐E. The Mann–Whitney *U* test is used to compare distribution means between ASD and TD groups, with asterisks indicating statistical significance. Gray EEG leads on the topographic maps denote channels that are part of statistically significant clusters.

Higher spindle amplitude and ISA were observed in the 10–16 Hz frequency range in children on the autism spectrum (Figure 2, detailed results in Supplementary Table S3). Specifically, we found a statistically significant increase in amplitude in a cluster composed of bilateral frontal and temporal electrodes (Figure 2, top amplitude boxplots, *U* = 958, *p* < 0.01). Moreover, we found increased ISA in a cluster composed of centro‐frontal electrodes and the right frontal F8 electrode (Figure 2, top ISA boxplots, *U* = 947, *p* < 0.01). We did not find statistically significant differences when comparing Density, Duration, and Frequency spindle parameters.

As part of a purely descriptive and exploratory analysis, we examined the distribution of spindle amplitude and ISA values across ASD subgroups. Interestingly, after averaging the absolute values within the significant clusters, we found a gradient of spindle amplitudes increasing with the severity of the clinical condition: lower in the ASDo group, intermediate in the ASD‐GDD, and higher in ASD‐GDD‐E (Figure 2, bottom amplitude boxplots, ASDo amplitude mean ± SD:21.44 ± 6.28 μV; ASD‐GDD amplitude mean ± SD:23.63 ± 5.28 μV, ASD‐GDD‐E amplitude mean ± SD:27.96 ± 6.12 μV). On the other hand, ISA values remained similar across subgroups (Figure 2, bottom ISA boxplots, ASDo ISA mean ± SD:8.21 ± 6.12 μV; ASD‐GDD ISA mean ± SD:9.16 ± 7.83 μV, ASD‐GDD‐E ISA mean ± SD:11.53 ± 7.63 μV).

### 3.6 Comparisons of Sleep Spindles Calculated in Three Frequency Sub‐Ranges

**FIGURE 3:**
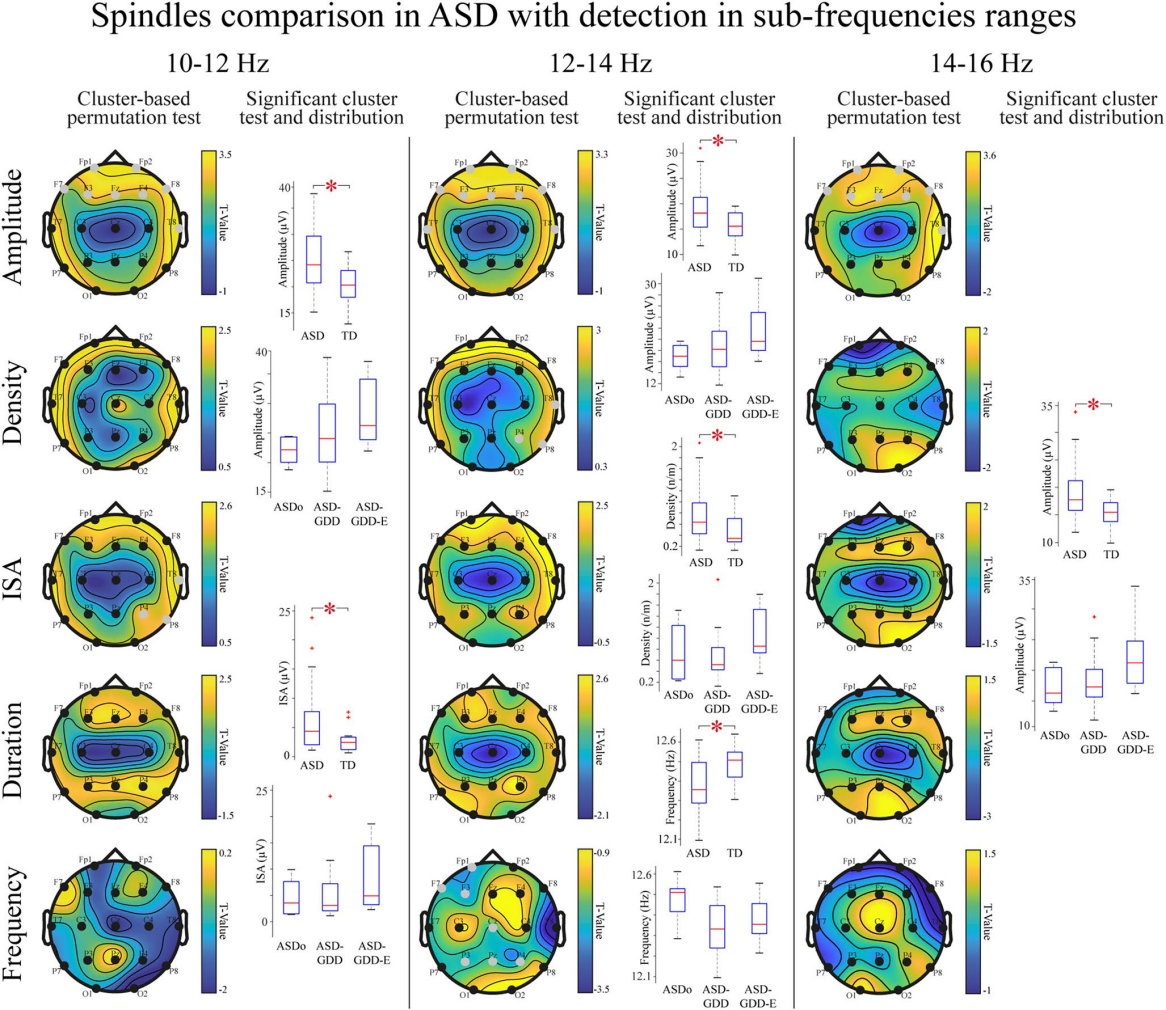
Spindle parameters in children on the autism spectrum detected in sub‐frequency ranges. Left panel, 10–12 Hz frequency range comparisons. In the left column, each row displays topographic maps showing *T*‐values derived from *t*‐tests for independent samples, applied within a cluster‐based permutation test to compare spindle parameters detected within the 10–12 Hz frequency range for typically developing (TD) and autism spectrum disability (ASD) children. The parameters analyzed from top to bottom are Amplitude, Density, Integrated Spindle Activity (ISA), Duration, and Frequency. The right column highlights the two parameters that showed significant differences in the cluster‐based permutation test: Amplitude and ISA. For these two parameters, boxplots illustrate the distribution of mean values averaged within the statistically significant clusters. Additionally, under each of these distributions three descriptive boxplots display the distribution of mean values within the same clusters among ASD subgroups, specifically ASDo, ASD‐GDD, and ASD‐GDD‐E. Central panel, 12–14 Hz frequency range comparisons. Similar to the left panels, but showing parameters detected in the 12–14 Hz frequency range. In this case, relevant parameters and subsequent showed distributions refer to Amplitude, Density and Frequency. Right panel, 14–16 Hz frequency range comparisons. Similar to the other panels, but showing parameters detected in the 14–16 Hz frequency range. In this case, the Amplitude parameter showed statistically significant differences in the comparison. Mann–Whitney *U* test is used to compare distribution means between ASD and TD groups, with asterisks indicating statistical significance. Gray EEG leads on the topographic maps denote channels that are part of statistically significant clusters.

Additional analyses on spindle parameters detected at 10–12, 12–14, and 14–16 Hz sub‐ranges showed the presence of different EEG electrode clusters between children on the autism spectrum in respect to TD children (Figure 3, detailed results in Table S4). A significant increase in amplitude was found in all band frequencies across the anterior electrode cluster only in ASD children (10–12 comparison: *U* = 948, *p* < 0.01; 12–14 comparison: *U* = 945, *p* < 0.01; 14–16 comparison: *U* = 962, *p* < 0.01). Density and frequency resulted increased in a posterior and a parieto‐centro‐left frontal cluster respectively in ASD children only in the 12–14 Hz frequency range (Density: *U* = 925, *p* = 0.03; Frequency: *U* = 674, *p* < 0.01). ISA were increased in ASD children in a right temporo‐parietal cluster only in the 10–12 Hz frequency range (*U* = 932, *p* = 0.02). Mean distributions of these statistically significant clusters are presented in Figure 3 as the top of each single parameter boxplot for both ASD and TD groups. We did not find statistically significant clusters when comparing the remaining spindle parameters.

The distribution of parameters identified as significantly different between the ASD and TD groups is also described across ASD subgroups for exploratory purposes (Figure 3, bottom top of each single parameter boxplot). Amplitude distribution of the cluster means across subgroups was similar to the distribution found in the broad‐band analysis. The ASDo group exhibited lower means (10–12 Hz mean ± SD:21.99 ± 4.85 μV; 12–14 Hz mean ± SD:16.76 ± 4.59 μV; 14–16 Hz Amplitude mean ± SD:17.06 ± 5.14 μV). The ASD‐GDD group exhibited intermediate mean values (10–12 Hz mean ± SD:24.93 ± 4.85 μV; 12–14 Hz mean ± SD:18.53 ± 4.49 μV; 14–16 Hz mean ± SD:18.31 ± 4.01 μV), and the ASD‐GDD‐E group displayed the higher means (10–12 Hz mean ± SD:28.69 ± 5.90 μV; 12–14 Hz mean ± SD:21.68 ± 5.18 μV; 14–16 Hz mean ± SD:22.41 ± 5.91 μV). A similar pattern with less separation between ASDo and ASD‐GDD was found in the density distribution for the cluster calculated in the 12–14 Hz analysis (ASDo density mean ± SD:0.74 ± 0.55n/m; ASD‐GDD density mean ± SD:0.68 ± 0.44n/m; ASD‐GDD‐E density mean ± SD:1.02 ± 0.54n/m), and in the ISA detected in the 10–12 Hz analysis (ASDo ISA mean ± SD:4.56 ± 3.83 μV; ASD‐GDD ISA mean ± SD:5.47 ± 5.48 μV; ASD‐GDD‐E ISA mean ± SD:8.54 ± 6.53 μV). Finally, the frequency parameters calculated at 12–14 Hz showed higher values for ASDo, lowest for ASD‐GDD, and intermediate for ASD‐GDD‐E (ASDo frequency mean ± SD:12.48 ± 0.17 Hz; ASD‐GDD frequency mean ± SD:12.33 ± 0.13 Hz; ASD‐GDD‐E frequency mean ± SD:12.37 ± 0.11 Hz).

## 4 Discussion

Our study investigated sleep spindle parameters in preschool‐aged children on the autism spectrum, both with and without the co‐occurrence of DD, during afternoon naps—a more practical approach within standard clinical settings. Using a standard 20‐electrode EEG system, we investigated variations in sigma power and spindle parameters to differentiate between children with ASD and TD peers. Concurrently, we conducted a detailed analysis of spindle parameters within specific frequency ranges and examined topographic differences. For descriptive and exploratory purposes only, we stratified ASD children into subgroups based on the possible co‐occurrence of Global Developmental Delay (GDD) or epileptic abnormalities.

In our study, individuals with ASD exhibited significant variations in both EEG sigma power and sleep spindle parameters, which were particularly pronounced in frontal/prefrontal electrodes. Specifically, we observed a prevalence of sigma power in frontal leads for the ASD group, whereas in TD individuals, it was more prominent in fronto‐central EEG leads. Additionally, we found increased power in the alpha and beta bands, which may reflect the varying developmental stages within our cohort. Periodic component peaks are known to occur at different frequencies across the lifespan, particularly during developmental stages (Kurth et al. 2010). Hence, it is possible that the sleep spindle frequency window in our sample was broader than the typical sigma band range. This underscores the importance of detecting spindles across a broader frequency window, as well as within the three sub‐frequency ranges, as demonstrated in this study.

Additionally, spindle parameters in the ASD group exhibited significant increases in amplitude and ISA over the anterior electrode cluster. Of note, the heightened amplitude in the anterior EEG leads was consistently observed in both ASD subgroups with GDD (ASD‐GDD and ASD‐GDD‐E). Our findings on spindle density align with previous work, which identified increased spindle density as a distinguishing feature between ASD and TD (Martinez and Chen 2023). However, the higher spindle amplitude observed in our ASD sample contrasts with previous reports that found no statistically significant differences in spindle amplitude between ASD and control groups (Martinez and Chen 2023; Cumming et al. 2024). This discrepancy may be attributed to differences in cohort composition. In Martinez and Chen's study, the age range of participants was 8–16 years, while our study focused on a younger cohort aged 2–5 years.

Age is known to influence thalamocortical synchrony and spindle morphology, which may account for the divergent results. Cumming et al. acknowledged that their methodology involved measuring spindle metrics separately for N2 fast and slow spindles throughout the entire night using an automated algorithm, resulting in findings that contrast with previous studies, including one based on the same dataset (Farmer et al. 2018). Additionally, they employed a different statistical approach by excluding the DD cohort from their primary analyses and including sex and race as covariates. Furthermore, our study provides additional insights into the regional specificity of these abnormalities, emphasizing the importance of anterior EEG leads in understanding sleep‐related differences in ASD.

Anomalous spindle morphology has been established as an indicator of psychomotor delay and intellectual disability (Shibagaki et al. 1982). Several studies also suggest an evolutionary trajectory of spindles during childhood (Campbell and Feinberg 2016; Novelli et al. 2016; Piantoni et al. 2016), which may differ substantially in Neurodevelopmental Disorders. However, the available data have yielded conflicting results (Farmer et al. 2018; Fletcher et al. 2020; Godbout et al. 2000; Limoges et al. 2005; Shibagaki et al. 1982). The existing literature is notably limited in ASD, where data predominantly pertain to high‐functioning subjects with non‐homogeneous age groups (Godbout et al. 1998; Tani et al. 2004; Tessier et al. 2015). Moreover, studies frequently diverge in the modalities of EEG recording, analysis algorithms, and the variables considered for sample selection (e.g., age, sex, comorbidity, presence of sleep disorders, drug therapy). These variations inevitably introduce a degree of variability in the results, making cross‐literature comparisons challenging. Our work underscores the complexity and heterogeneity of sleep spindle parameters in preschool‐aged children on the autism spectrum.Of note, the subgroup comparisons presented in this study were not subjected to inferential statistical testing and should be interpreted with caution. They are intended solely to generate hypotheses for future research. Nonetheless, the consistent elevation in frontal spindle amplitude across all ASD cases with GDD co‐occurrence may suggest a relationship between spindle characteristics and GDD. Further studies with larger, stratified samples are needed to determine whether these patterns reflect meaningful subgroup differences or are instead driven by associated developmental features. Our study provides novel perspectives in the current literature, which often lacks comprehensive studies integrating diverse methodologies and demographic variables. Further investigations with standardized EEG protocols and longitudinal designs are warranted to elucidate the developmental trajectories of sleep spindles in children on the autism spectrum and their implications for early intervention strategies.

The reported findings suggest that developmental changes in spindle characteristics that are detectable from EEG frontal leads may reflect substantial neurobiological alterations that play a crucial role in cognitive and socio‐communicative functioning. The frontal EEG predominance of sigma power in the ASD group and fronto‐central in the TD group implies a delay in the maturation of spindles along the antero‐posterior axis (D'Atri et al. 2018; Novelli et al. 2016). Furthermore, the presence of spindles with higher amplitude and density recalls the “extreme spindles” previously described by Gibbs and Bixler (Bixler and Rhodes 1968; Gibbs and Gibbs 1962) in children with impaired cognitive development.

Our study has several limitations. Importantly, scalp recordings do not allow direct inference about the exact neural generators of the observed activity. Thus, while our findings consistently highlight specific EEG leads, the underlying sources may involve distributed and/or deeper structures not directly accessible through surface electrodes. Moreover, the sample size was relatively small, and there was a gender difference between groups, with a higher percentage of females in the TD group. Although δ power decreases earlier in developing females, perhaps reflecting earlier synaptic pruning compared to males, differences in spindle activity between sexes are generally limited in prepuberal children, especially with neurodevelopmental disorders (Zhang et al. 2021). Additionally, methodological differences, such as the acquisition of nap PSG, make our results challenging to compare with previous literature. As shown by Bódizs et al. in 2021, nap sleep spindles are faster than nocturnal sleep spindles. Nevertheless, existing evidence indicates a deceleration in sleep spindles during the night (Bódizs et al. 2021). Consequently, our comparison may be more appropriately construed as analogous to an early‐night sleep cycle comparison rather than a late‐night sleep cycle comparison. Fortunately, this is particularly advantageous for conditions characterized by fragmented physiological sleep, such as neurodevelopmental disorders. Moreover, nap PSG offers several potential advantages, being an easier, less expensive, and more acceptable approach for children with behavioral difficulties. Furthermore, behavioral interventions to regularize circadian rhythms were not carried out before PSG acquisition.

One final limitation of our study is the potential underestimation of sigma activity due to possibly lower and variable individual periodic rhythms in our young pediatric cohort. The literature, including works by Kurth et al. (2010) and Perinelli et al. (2022), highlights that the variability in EEG regionality and peak frequency associated with developmental age can significantly impact the accuracy of frequency band definitions. The calculation of Individual Alpha Frequency, for example, resulted particularly challenging in naps from young children, where alpha peaks are known to be reduced during sleep EEG and strong individual variability of periodic peak is present. We recognize that this quantification is crucial for further interpreting our findings and underscores the necessity for future research on new methodologies that can more accurately capture EEG periodic components in pediatric populations.

Combined, our findings corroborate the diagnostic potential of sleep spindle parameters in the ASD group and confirm that afternoon naps are sufficient to identify sleep spindle abnormalities in children. Although it is well established that spindle frequency changes with developmental age (Campbell and Feinberg 2016; Shinomiya et al. 1999; Tarokh et al. 2010), recent large‐scale studies have shown that this trajectory is non‐linear (Kwon et al. 2023) and may involve both increases and decreases depending on spindle type (Kozhemiako et al. 2024). Moreover, there is a lack of data on the developmental trajectory of spindle frequency in children on the autism spectrum. Our results show alterations in spindle parameters that may reflect dysregulated maturation of thalamocortical circuitry, underscoring the importance of continued exploration in this domain.

## Author Contributions

**Sasha D’Ambrosio:** drafting and data analysis. **Daniele Gualandris and Davide Caputo:** data collection and analysis. **Alessia Mingarelli and Federico Raviglione:** data collection. **Francesco Donati, Ahmad Mayeli, and Renata del Giudice:** data analysis. **Fabio Ferrarelli, Maria Paola Canevini, and Armando D’Agostino:** critical review of the manuscript, resources, and study design.

## Ethics Statement

In conducting this study, we adhered to the principles outlined in the Declaration of Helsinki regarding the ethical conduct of research involving human subjects. All procedures were performed in accordance with these principles to ensure the protection of participants' rights, confidentiality, and welfare.

## Conflicts of Interest

The authors declare no conflicts of interest.

## Supporting information


Data S1.

## Data Availability

The data that support the findings of this study are available on request from the corresponding author. The data are not publicly available due to privacy or ethical restrictions.

## References

[aur70087-bib-0001] Bang, J. W. , O. Khalilzadeh , M. Hämäläinen , T. Watanabe , and Y. Sasaki . 2014. “Location Specific Sleep Spindle Activity in the Early Visual Areas and Perceptual Learning.” Vision Research 99: 162–171. 10.1016/j.visres.2013.12.014. 24380705 PMC4041809

[aur70087-bib-0002] Bixler, E. O. , and J. M. Rhodes . 1968. “Spindle Activity During Sleep in Cultural‐Familial Mild Retardates.” Psychophysiology 5: 212.

[aur70087-bib-0003] Bódizs, R. , O. Szalárdy , C. Horváth , et al. 2021. “A Set of Composite, Non‐Redundant EEG Measures of NREM Sleep Based on the Power Law Scaling of the Fourier Spectrum.” Scientific Reports 11: 2041. 10.1038/s41598-021-81230-7. 33479280 PMC7820008

[aur70087-bib-0004] Campbell, I. G. , and I. Feinberg . 2016. “Maturational Patterns of Sigma Frequency Power Across Childhood and Adolescence: A Longitudinal Study.” Sleep 39: 193–201. 10.5665/sleep.5346. 26285004 PMC4678354

[aur70087-bib-0005] Chatburn, A. , S. Coussens , K. Lushington , D. Kennedy , M. Baumert , and M. Kohler . 2013. “Sleep Spindle Activity and Cognitive Performance in Healthy Children.” Sleep 36: 237–243. 10.5665/sleep.2380. 23372271 PMC3543056

[aur70087-bib-0006] Clawson, B. C. , J. Durkin , and S. J. Aton . 2016. “Form and Function of Sleep Spindles Across the Lifespan.” Neural Plasticity 2016: e6936381. 10.1155/2016/6936381. PMC4848449 27190654

[aur70087-bib-0008] Cumming, D. , N. Kozhemiako , A. E. Thurm , C. A. Farmer , S. Purcell , and A. W. Buckley . 2024. “Spindle Chirp and Other Sleep Oscillatory Features in Young Children With Autism.” Sleep Medicine 119: 320–328. 10.1016/j.sleep.2024.05.008. 38733760 PMC11348284

[aur70087-bib-0056] D’Agostino, A. , A. Castelnovo , S. Cavallotti . et al. 2018. “Sleep Endophenotypes of Schizophrenia: Slow Waves and Sleep Spindles in Unaffected First‐Degree Relatives.” Npj Schizophr 4: 2. 10.1038/s41537-018-0045-9. 29426848 PMC5807540

[aur70087-bib-0009] D'Atri, A. , L. Novelli , M. Ferrara , O. Bruni , and L. De Gennaro . 2018. “Different Maturational Changes of Fast and Slow Sleep Spindles in the First Four Years of Life.” Sleep Medicine 42: 73–82. 10.1016/j.sleep.2017.11.1138. 29458750

[aur70087-bib-0010] Delorme, A. , and S. Makeig . 2004. “EEGLAB: An Open Source Toolbox for Analysis of Single‐Trial EEG Dynamics Including Independent Component Analysis.” Journal of Neuroscience Methods 134: 9–21. 10.1016/j.jneumeth.2003.10.009. 15102499

[aur70087-bib-0011] Diagnostic and statistical manual of mental disorders: DSM‐5, 5th ed . 2013. Diagnostic and Statistical Manual of Mental Disorders: DSM‐5. 5th ed. American Psychiatric Publishing, Inc. 10.1176/appi.books.9780890425596.

[aur70087-bib-0012] Farmer, C. A. , P. Chilakamarri , A. E. Thurm , S. E. Swedo , G. L. Holmes , and A. W. Buckley . 2018. “Spindle Activity in Young Children With Autism, Developmental Delay, or Typical Development.” Neurology 91: e112–e122. 10.1212/WNL.0000000000005759. 29875224 PMC6053112

[aur70087-bib-0013] Ferrarelli, F. , M. Massimini , S. Sarasso , et al. 2010. “Breakdown in Cortical Effective Connectivity During Midazolam‐Induced Loss of Consciousness.” Proceedings of the National Academy of Sciences of the United States of America 107: 2681–2686. 10.1073/pnas.0913008107. 20133802 PMC2823915

[aur70087-bib-0014] Ferrarelli, F. , S. Sarasso , Y. Guller , et al. 2012. “Reduced Natural Oscillatory Frequency of Frontal Thalamocortical Circuits in Schizophrenia.” Archives of General Psychiatry 69: 766–774. 10.1001/archgenpsychiatry.2012.147. 22474071 PMC3394893

[aur70087-bib-0015] Fletcher, F. E. , V. Knowland , S. Walker , M. G. Gaskell , C. Norbury , and L. M. Henderson . 2020. “Atypicalities in Sleep and Semantic Consolidation in Autism.” Developmental Science 23: e12906. 10.1111/desc.12906. 31569286 PMC7187235

[aur70087-bib-0016] Fogel, S. M. , R. Nader , K. A. Cote , and C. T. Smith . 2007. “Sleep Spindles and Learning Potential.” Behavioral Neuroscience 121: 1–10. 10.1037/0735-7044.121.1.1. 17324046

[aur70087-bib-0017] Geiger, A. , R. Huber , S. Kurth , M. Ringli , O. G. Jenni , and P. Achermann . 2011. “The Sleep EEG as a Marker of Intellectual Ability in School Age Children.” Sleep 34: 181–189. 10.1093/sleep/34.2.181. 21286251 PMC3022938

[aur70087-bib-0018] Gibbs, E. L. , and F. A. Gibbs . 1962. “Extreme Spindles: Correlation of Electroencephalographic Sleep Pattern With Mental Retardation.” Science 138: 1106–1107. 10.1126/science.138.3545.1106. 13947675

[aur70087-bib-0019] Godbout, R. , C. Bergeron , E. Limoges , E. Stip , and L. Mottron . 2000. “A Laboratory Study of Sleep in Asperger's Syndrome.” Neuroreport 11: 127–130. 10.1097/00001756-200001170-00025. 10683843

[aur70087-bib-0020] Godbout, R. , C. Bergeron , E. Stip , and L. Mottron . 1998. “A Laboratory Study of Sleep and Dreaming in a Case of Asperger's Syndrome.” Dreaming 8: 75–88. 10.1023/B:DREM.0000005898.95212.58.

[aur70087-bib-0021] Gonzalez, C. , X. Jiang , J. Gonzalez‐Martinez , and E. Halgren . 2022. “Human Spindle Variability.” Journal of Neuroscience 42: 4517–4537. 10.1523/JNEUROSCI.1786-21.2022. 35477906 PMC9172080

[aur70087-bib-0022] Gonzalez, C. E. , R. A. Mak‐McCully , B. Q. Rosen , et al. 2018. “Theta Bursts Precede, and Spindles Follow, Cortical and Thalamic Downstates in Human NREM Sleep.” Journal of Neuroscience 38: 9989–10001. 10.1523/JNEUROSCI.0476-18.2018. 30242045 PMC6234298

[aur70087-bib-0023] Gorgoni, M. , S. Scarpelli , F. Reda , and L. De Gennaro . 2020. “Sleep EEG Oscillations in Neurodevelopmental Disorders Without Intellectual Disabilities.” Sleep Medicine Reviews 49: 101224. 10.1016/j.smrv.2019.101224. 31731102

[aur70087-bib-0024] Griffiths, R. 1970. The Abilities of Young Children: A Comprehensive System of Mental Measurement for the First Eight Years of Life by Ruth Griffiths: Fair (1970). Child Development Research Centre. https://www.abebooks.com/Abilities‐Young‐Children‐Comprehensive‐System‐Mental/30971558314/bd.

[aur70087-bib-0025] Gruber, R. , G. Somerville , L. Bergmame , L. Fontil , and S. Paquin . 2016. “School‐Based Sleep Education Program Improves Sleep and Academic Performance of School‐Age Children.” Sleep Medicine 21: 93–100. 10.1016/j.sleep.2016.01.012. 27448478

[aur70087-bib-0026] Huber, R. , T. Graf , K. A. Cote , et al. 2000. “Exposure to Pulsed High‐Frequency Electromagnetic Field During Waking Affects Human Sleep EEG.” Neuroreport 11: 3321–3325. 10.1097/00001756-200010200-00012. 11059895

[aur70087-bib-0027] Kaskie, R. E. , K. M. Gill , and F. Ferrarelli . 2019. “Reduced Frontal Slow Wave Density During Sleep in First‐Episode Psychosis.” Schizophrenia Research 206: 318–324. 10.1016/j.schres.2018.10.024. 30377012

[aur70087-bib-0055] Kawahara, M. , K. Kagitani‐Shimono , K. Kato‐Nishimura , et al. 2022. “A Preliminary Study of Sleep Spindles Across Non‐Rapid Eye Movement Sleep Stages in Children with Autism Spectrum Disorder.” Sleep Advances 3: 1–11. 10.1093/sleepadvances/zpac037. PMC10104411 37193405

[aur70087-bib-0028] Kozhemiako, N. , A. W. Buckley , R. D. Chervin , S. Redline , and S. M. Purcell . 2024. “Mapping Neurodevelopment With Sleep Macro‐ and Micro‐Architecture Across Multiple Pediatric Populations.” NeuroImage: Clinical 41: 103552. 10.1016/j.nicl.2023.103552. 38150746 PMC10788305

[aur70087-bib-0029] Kurth, S. , M. Ringli , A. Geiger , M. LeBourgeois , O. G. Jenni , and R. Huber . 2010. “Mapping of Cortical Activity in the First Two Decades of Life: A High‐Density Sleep Electroencephalogram Study.” Journal of Neuroscience 30: 13211–13219. 10.1523/JNEUROSCI.2532-10.2010. 20926647 PMC3010358

[aur70087-bib-0030] Kurth, S. , M. Ringli , M. K. LeBourgeois , et al. 2012. “Mapping the Electrophysiological Marker of Sleep Depth Reveals Skill Maturation in Children and Adolescents.” NeuroImage 63: 959–965. 10.1016/j.neuroimage.2012.03.053. 22498654 PMC4444061

[aur70087-bib-0031] Kurz, E.‐M. , A. Conzelmann , G. M. Barth , et al. 2019. “Signs of Enhanced Formation of Gist Memory in Children With Autism Spectrum Disorder—A Study of Memory Functions of Sleep.” Journal of Child Psychology and Psychiatry 60: 907–916. 10.1111/jcpp.13048. 30908649 PMC6850042

[aur70087-bib-0032] Kwon, H. , K. G. Walsh , E. D. Berja , et al. 2023. “Sleep Spindles in the Healthy Brain From Birth Through 18 Years.” Sleep 46: zsad017. 10.1093/sleep/zsad017. 36719044 PMC10091086

[aur70087-bib-0033] Limoges, É. , L. Mottron , C. Bolduc , C. Berthiaume , and R. Godbout . 2005. “Atypical Sleep Architecture and the Autism Phenotype.” Brain 128: 1049–1061. 10.1093/brain/awh425. 15705609

[aur70087-bib-0034] Lustenberger, C. , A.‐L. Mouthon , N. Tesler , et al. 2017. “Developmental Trajectories of EEG Sleep Slow Wave Activity as a Marker for Motor Skill Development During Adolescence: A Pilot Study.” Developmental Psychobiology 59: 5–14. 10.1002/dev.21446. 27401676

[aur70087-bib-0035] Lustenberger, C. , F. Wehrle , L. Tüshaus , P. Achermann , and R. Huber . 2015. “The Multidimensional Aspects of Sleep Spindles and Their Relationship to Word‐Pair Memory Consolidation.” Sleep 38: 1093–1103. 10.5665/sleep.4820. 25845686 PMC4481015

[aur70087-bib-0036] Martinez, C. , and Z. S. Chen . 2023. “Identification of Atypical Sleep Microarchitecture Biomarkers in Children With Autism Spectrum Disorder.” Frontiers in Psychiatry 14: 1115374. 10.3389/fpsyt.2023.1115374. 37139324 PMC10150704

[aur70087-bib-0038] Mylonas, D. , S. Machado , O. Larson , et al. 2022. “Dyscoordination of Non‐Rapid Eye Movement Sleep Oscillations in Autism Spectrum Disorder.” Sleep 45: zsac010. 10.1093/sleep/zsac010. 35022792

[aur70087-bib-0039] Novelli, L. , A. D'atri , C. Marzano , et al. 2016. “Mapping Changes in Cortical Activity During Sleep in the First 4 Years of Life.” Journal of Sleep Research 25: 381–389. 10.1111/jsr.12390. 26854271

[aur70087-bib-0040] Oostenveld, R. , P. Fries , E. Maris , and J.‐M. Schoffelen . 2011. “FieldTrip: Open Source Software for Advanced Analysis of MEG, EEG, and Invasive Electrophysiological Data.” Computational Intelligence and Neuroscience 2011: 156869. 10.1155/2011/156869. 21253357 PMC3021840

[aur70087-bib-0041] Page, J. , C. Lustenberger , and F. Fröhlich . 2018. “Social, Motor, and Cognitive Development Through the Lens of Sleep Network Dynamics in Infants and Toddlers Between 12 and 30 Months of Age.” Sleep 41: zsy024. 10.1093/sleep/zsy024. 29506060 PMC6018907

[aur70087-bib-0042] Page, J. , C. Lustenberger , and F. Fröhlich . 2020. “Nonrapid Eye Movement Sleep and Risk for Autism Spectrum Disorder in Early Development: A Topographical Electroencephalogram Pilot Study.” Brain and Behavior: A Cognitive Neuroscience Perspective 10: e01557. 10.1002/brb3.1557. PMC7066345 32037734

[aur70087-bib-0043] Perinelli, A. , S. Assecondi , C. F. Tagliabue , and V. Mazza . 2022. “Power Shift and Connectivity Changes in Healthy Aging During Resting‐State EEG.” NeuroImage 256: 119247. 10.1016/j.neuroimage.2022.119247. 35477019

[aur70087-bib-0044] Piantoni, G. , E. Halgren , and S. S. Cash . 2016. “The Contribution of Thalamocortical Core and Matrix Pathways to Sleep Spindles.” Neural Plasticity 2016: e3024342. 10.1155/2016/3024342. PMC4842069 27144033

[aur70087-bib-0046] Shibagaki, M. , S. Kiyono , and K. Watanabe . 1982. “Spindle Evolution in Normal and Mentally Retarded Children: A Review.” Sleep 5: 47–57. 10.1093/sleep/5.1.47. 7071451

[aur70087-bib-0047] Shinomiya, S. , K. Nagata , K. Takahashi , and T. Masumura . 1999. “Development of Sleep Spindles in Young Children and Adolescents.” Clinical Electroencephalography 30: 39–43. 10.1177/155005949903000203. 10358781

[aur70087-bib-0048] Silber, M. H. , S. Ancoli‐Israel , M. H. Bonnet , et al. 2007. “The Visual Scoring of Sleep in Adults.” Journal of Clinical Sleep Medicine 3: 121–131. 17557422

[aur70087-bib-0049] Tani, P. , N. Lindberg , T. Nieminen‐von Wendt , et al. 2004. “Sleep in Young Adults With Asperger Syndrome.” Neuropsychobiology 50: 147–152. 10.1159/000079106. 15292669

[aur70087-bib-0050] Tarokh, L. , M. A. Carskadon , and P. Achermann . 2010. “Developmental Changes in Brain Connectivity Assessed Using the Sleep EEG.” Neuroscience 171: 622–634. 10.1016/j.neuroscience.2010.08.071. 20833232 PMC4119998

[aur70087-bib-0051] Tessier, S. , A. Lambert , M. Chicoine , P. Scherzer , I. Soulières , and R. Godbout . 2015. “Intelligence Measures and Stage 2 Sleep in Typically‐Developing and Autistic Children.” International Journal of Psychophysiology 97: 58–65. 10.1016/j.ijpsycho.2015.05.003. 25958790

[aur70087-bib-0052] Timofeev, I. , and S. Chauvette . 2013. “The Spindles: Are They Still Thalamic?” Sleep 36: 825–826. 10.5665/sleep.2702. 23729924 PMC3649824

[aur70087-bib-0053] Wechsler, D. 2012. “Wechsler Intelligence Scale for Children, Fourth Edition.” 10.1037/t15174-000.

[aur70087-bib-0054] Zhang, Z. Y. , I. G. Campbell , P. Dhayagude , H. C. Espino , and I. Feinberg . 2021. “Longitudinal Analysis of Sleep Spindle Maturation From Childhood Through Late Adolescence.” Journal of Neuroscience 41: 4253–4261. 10.1523/JNEUROSCI.2370-20.2021. 33785642 PMC8143202

