# Supplementary material for: Sleep Spindle Abnormalities in Preschool Children With Autism Spectrum Disability: Insights From Nap Polysomnography
Source: Autism Res. 2025 Jul 23;18(9):1764–74. doi: 10.1002/aur.70087 (PMC12442524; doi:10.1002/aur.70087)
Supplement: Supplementary file 1 — Data S1. [file AUR-18-1764-s001.docx]

Supplementary Figure 1:


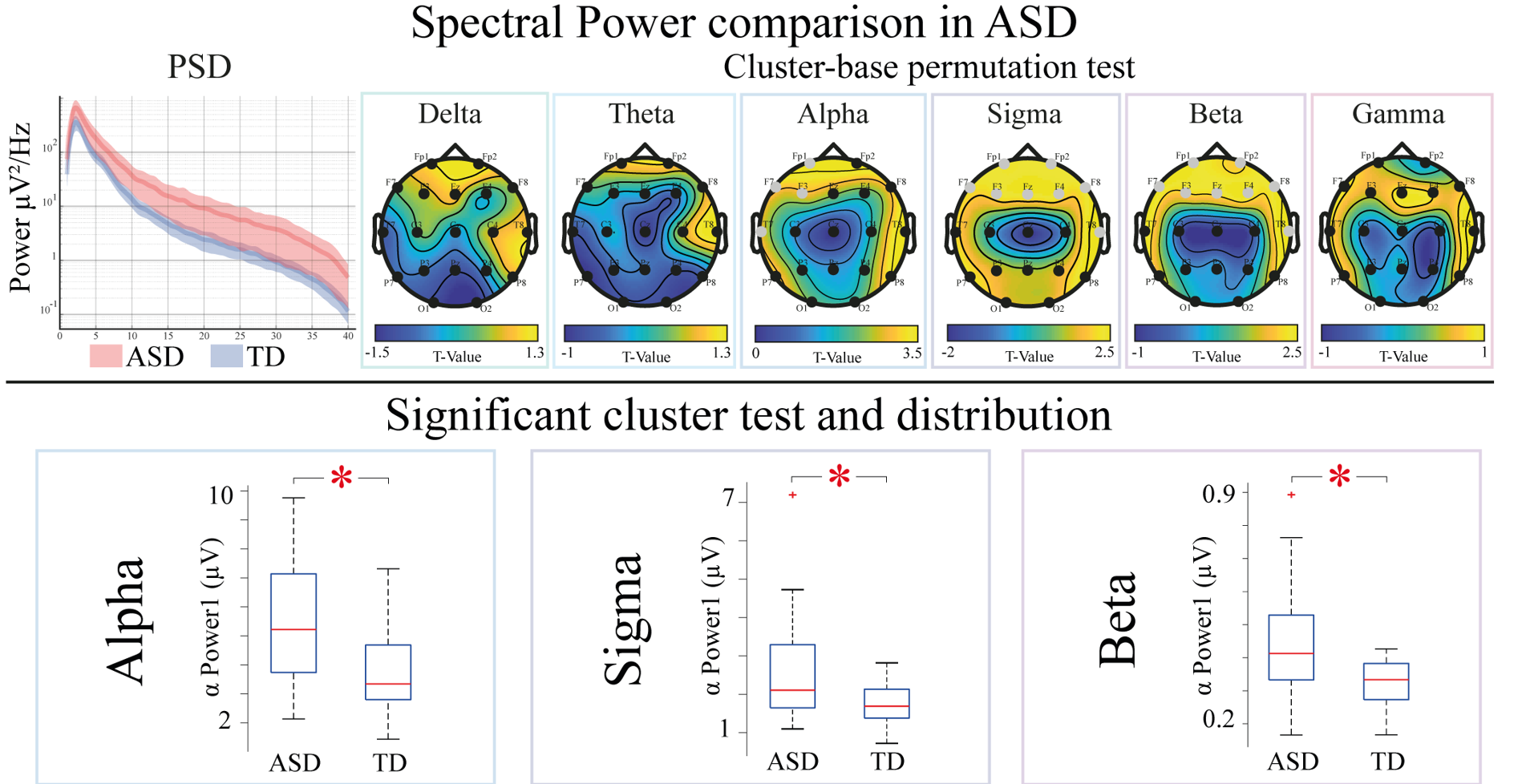

**Supplementary Figure 1.** Broad band spectral power comparison in children on the autism spectrum. Top left. PSD (Power Spectral Density): Comparison of power spectral density between children on the autism spectrum (ASD, light red) and typically developing (TD, light blue) children across a frequency range of 0 to 40 Hz. The power spectrum shows the mean spectral power averaged across all channels and subjects within the two groups. Shaded areas around the curves denote the standard error of the mean. Top centre and top right. Topographic distribution of the T-values derived from the t-test for independent samples used in a cluster-based permutation test to compare ASD and TD groups for EEG power in six frequency bands. Namely, from left to right, Delta, Theta, Alpha, Sigma, Beta, and Gamma. Bottom panels. Boxplot displays the distribution of mean values averaged within the statistically significant clusters identified. Specifically, statistically significant clusters were identified when comparing Alpha (left), Sigma (centre), and Beta (right) EEG bands. In all panels, the Mann-Whitney U test is used to compare distribution means, with asterisks indicating statistical significance. Gray EEG leads on the topographic maps denote channels that are part of statistically significant clusters.

Supplementary Table 1:

**Supplementary Table 1.** Individual information for all participants included in this study, including gender, age, diagnosis subtype (Children with ASD diagnosis only (ASDo); Children with ASD and General Developmental Delay (GDD) co-occurrence (ASD-GDD); Children with ASD, GDD and EEG epileptic abnormalities co-occurrences (ASD-GDD-E), without clinical history of epilepsy and free from EEG seizures; Typically developed children (TD)). The table also reports the presence of EEG abnormalities, sleep disorders, and neuroimaging (MRI) findings.

Supplementary Table 2:


**Supplementary Table 2.** Detailed results of the comparisons between ASD and TD EEG powers that resulted significant: delta (1-4.5 Hz); theta (4.5-8 Hz); alpha (8-11.5 Hz); sigma (11.5-15.5 Hz) high beta (15-25 Hz); and gamma (25-40 Hz) comparisons. For each band, the table reports EEG power mean and standard deviation of the cluster resulted statistically significant in ASD and TD groups. For descriptive purposes, this information is reported also for the ASDo, ASD-GDD and ASD-GDD-E subgroups. Moreover, for the comparisons between ASD and TD, this table present the list of electrodes involved in the statistically significant clusters and their corresponding effect size and power.

Supplementary Table 3:


**Supplementary Table 3.** Detailed results of the comparisons between ASD and TD sleep spindles parameters detected between 10 and 16Hz (broadband analysis). This table presents the mean and standard deviation of the Amplitude, Density, Intrinsic Spindles Activity (ISA), Duration and Frequency parameters for each electrode across all subjects for the two comparative group ASD and TD respectively. Moreover, for each parameter in which a statistically significant different EEG cluster has been detected, mean and standard deviation are presented for the ASD and TD groups. For descriptive purposes, this information is reported also for the ASDo, ASD-GDD and ASD-GDD-E subgroups. Finally, for the comparisons between ASD and TD, this table reports the list of electrodes included in each statistically significant cluster, along with the corresponding effect sizes and statistical power for each parameter.

Supplementary Table 4:


**Supplementary Table 4.** Detailed results of the comparisons between ASD and TD groups for sleep spindle parameters detected within the following ranges: 10–12 Hz (left), 12–14 Hz (center), and 14–16 Hz (right). For each sub-frequency analysis, this table presents the mean and standard deviation of the Amplitude, Density, Intrinsic Spindles Activity (ISA), Duration and Frequency parameters for each electrode across all subjects for the two comparative group ASD and TD respectively. Moreover, for each parameter in which a statistically significant different EEG cluster has been detected, mean and standard deviation are presented for the ASD and TD groups. For descriptive purposes, this information is reported also for the ASDo, ASD-GDD and ASD-GDD-E subgroups. Finally, for the comparisons between ASD and TD, are presented the list of electrodes included in each statistically significant cluster, along with the corresponding effect sizes and statistical power for each parameter.
